# Disruption of LRRK2 Does Not Cause Specific Loss of Dopaminergic Neurons in Zebrafish

**DOI:** 10.1371/journal.pone.0020630

**Published:** 2011-06-16

**Authors:** Guiqi Ren, Shengchang Xin, Song Li, Hanbing Zhong, Shuo Lin

**Affiliations:** 1 Laboratory of Chemical Genomics, School of Chemical Biology and Biotechnology, Peking University Shenzhen Graduate School, Shenzhen, China; 2 Department of Molecular, Cell and Developmental Biology, University of California Los Angeles, Los Angeles, California, United States of America; National Institutes of Health, United States of America

## Abstract

Mutations in LRRK2 are genetically linked to Parkinson's disease (PD) but its normal biological function is largely unknown. Sheng *et al.* recently reported that deletion of the WD40 domain of LRRK2 in zebrafish specifically causes PD-like loss of neurons and behavior defect. However, our similar early study and recent confirming experiments using the same reagents reported by Sheng *et al.* failed to reproduce the phenotype of the loss of dopaminergic neurons, although the mRNA of LRRK2 was molecularly disrupted. Our study suggests that function of LRRK2 and its usefulness to generate zebrafish PD model needs further evaluation.

## Introduction

Parkinson's disease (PD) is a common neurodegenerative disorder affecting approximately 1% of the population over the age of 50[1]. The primary symptoms of PD are movement dysfunctions, including tremor, rigidity, bradykinesia, and postural instability[2]. The pathologic hallmarks of PD are loss of dopaminergic (DA) neurons in the substantia nigra (SN) and the presence of Lewy bodies in the brain. Although most PD patients are idiopathic, 5–10% of PD patients are diagnosed to be linked to certain gene mutations, such as α-synuclein, UCHL1, LRRK2 (Leucine-rich repeat kinase 2), PINK1, Parkin, DJ-1, and ATP13A2[3]. Among these genes, LRRK2 represents the most prevalent genetic cause of autosomal-dominant PD[4], [5], [6], [7].

Human LRRK2 encodes a huge protein of 2527 amino acid and contains several functional domains including ARM (Armadillo), ANK (Ankyrin repeat), LRR (Leucine rich repeat), Roc (Ras of complex proteins, GTPase), COR (C-terminal of Roc), MAPKKK (Mitogen activated kinase kinase kinase), and WD40 from the N-terminus to the C-terminus. Over 40 point mutations have been identified in LRRK2, covering all of the functional domains, but proven pathogenic mutations appear concentrated in the GTPase and kinase domains[3], [8]. The most common pathogenic mutation is G2019S in the kinase domain, which is identified in ∼1% sporadic PD patients and ∼4% familial PD patients [9]. Overexpression of pathogenic variants of LRRK2 is toxic in cultured neuronal cells[10], [11]. *In vitro* kinase activity assay using moesin as substrate showed mutation G2019S increased the kinase activity of LRRK2, implying that the hyper kinase activity of LRRK2 is the cause of PD[12]. In transgenic mice, overexpression of mutant LRRK2 *^R1441G^* resulted in age-dependent and levodopa-responsive slowness of movement while overexpression of wild type LRRK2 did not cause typical symptom of PD [13]. In another study, overexpression of LRRK2 *^G2019S^* caused decreases in striatal dopamine content, release, and uptake[14]. However, the most characteristic feature of PD, loss of DA neurons, was not observed in these transgenic mice. Also, transgenic overexpession does not address the loss of function issue of LRRK2, which is critical for providing information of its normal biological function.

Zebrafish is a well established animal model for studying human diseases and has been used to investigate PD[15]. In addition, zebrafish embryos are susceptible to the treatment by the classic dopaminergic neurotoxin MPTP (1-Methyl-4-Phenyl-1,2,3,6-Tetrahydropyridine), which causes loss of DA neurons in zebrafish embryonic diencephalon, mimicking the key feature of PD [16], [17]. Recently, Sheng *et al*. reported a functional study of LRRK2 in zebrafish, showing that deletion of the WD40 domain of zebrafish LRRK2 produced typical PD phenotype including specific loss of DA neurons and locomotive defect without overall developmental defect[18]. We have also been investigating LRRK2 function in zebrafish prior to Sheng *et al*. 's publication but were not able to observe the reported phenotype. In more recent studies, we further confirmed that the DA neurons appear normal in embryos with depletion of LRRK2 mRNA by injection of the exact same morpholino oligos (MOs) reported by Sheng *et al*. Our studies suggest that function of LRRK2 and its usefulness to generate zebrafish PD model needs further evaluation.

## Results and Discussion

### Design and validation of MOs targeting LRRK2

**Figure 1:**
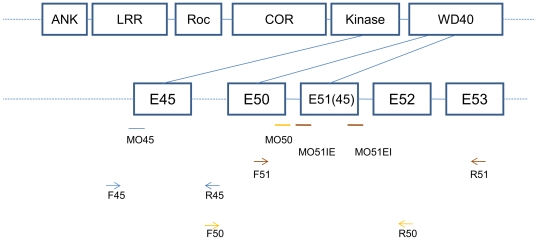
Schematic representation of the target sites of the morpholino oligos and the positions of the primers used in RT-PCR. The morpholino oligos target Exon 45 (E45), Exon 50 (E50) and Exon 51 (E51), which correspond to the coding sequences of Kinase, WD40 and WD40 domain respectively. Exon 51, shown as E51 (45), is the same as Exon 45 described by Sheng *et al*. The morpholino oligos and their corresponding primers are indicated by the same color.

Bioinformatics analysis of genome sequence revealed that zebrafish contains a single copy of highly conserved LRRK2 homolog (Ensembl ENSDARG00000006169) that has all of the functional domains[18]. We observed similar expression pattern of LRRK2 as reported by Sheng *et al*. To study the function of LRRK2 in zebrafish, three MOs (MO45, MO50, and MO51IE') were designed to disrupt the splicing of kinase and WD40 domains of LRRK2 (Fig. 1). To determine whether these three MOs did not produce the reported phenotype due to the difference of targeting sites, the two exact RNA splice-blocking MOs (IE and EI) used by Sheng *et al*. were tested and re-named as MO51IE and MO51EI, respectively in this paper. Our MO51IE' is one nucleotide upstream to MO51IE and to make comparison concise, the data of MO51IE' is not shown.

**Figure 2:**
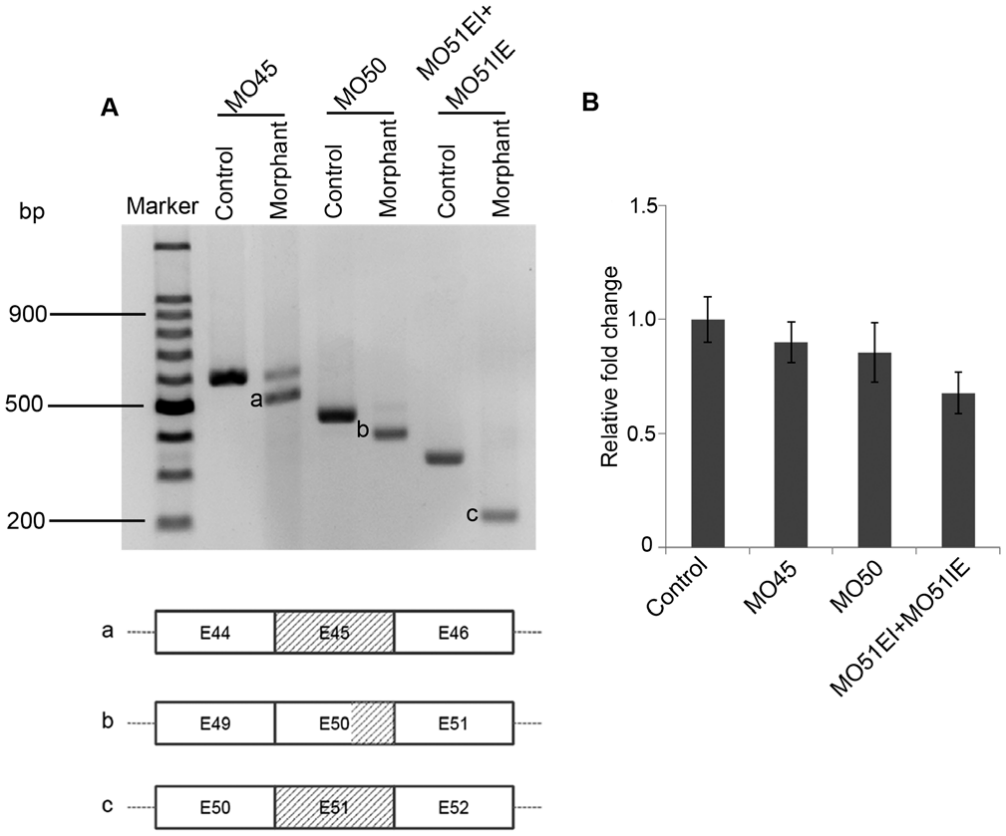
The splicing of lrrk2 mRNA was interrupted by the morpholino oligos. The injection dose of the morpholino oligos was 8 ng each per embryo. A, RT-PCR with cDNA of 3 dpf embryos (control and morphant) confirmed the blockage of normal splicing. The aberrant splice products (band a, b and c in A) were cloned and sequenced.The slash lines indicate the deletion of exons. All of the aberrant mRNA variants have reading frame shifts and pre-mature stop codons, and produce putative truncated LRRK2 proteins in the corresponding domains shown in Figure 1. In the lanes of MO50 morphant and MO51EI+MO51IE morphant, almost no wild type LRRK2 mRNA was present. B, qPCR using primers flanking the region upstream to MO target sites shows at least 60% LRRK2 transcripts can be detected, indicating no obvious nonsense mediated decay of LRRK2. Data were shown as mean±S.E.M of biological triplicates from at least two independent experiments and were presented as fold changes in relative gene expression as compared with control after being normalized to beta-actin. The relative fold changes were calculated by a comparative C_t_ method. *P*>>0.05 between control, MO45, and MO50 group; *P* < 0.05 between control and MO51EI+MO51IE group.

The efficiency of these MOs was evaluated by RT-PCR with primers flanking the MO target sites at 3 dpf (days post fertilization) embryos (Fig. 1). At a dose of 8 ng per embryo, all MOs were able to block the normal splicing of zebrafish LRRK2, leading to exon deletion (Fig. 2A, bands a to c). Co-injection of MO51IE+MO51EI resulted in dramatic decrease of wild type LRRK2 transcript (Fig. 2A, band c), confirming the efficacy of molecular knockdown of LRRK2 mRNA by MOs used by Sheng *et al*. To further determine whether there was drastic nonsense mediated decay of LRRK2 transcripts, a quantitative PCR test was performed using primers flanking region 5′-upstream to MO target sites, which are as same as Sheng *et al*. used, and no obvious decay of LRRK2 transcripts was detected in morphant groups, at least 60% LRRK2 transcripts remaining (Fig. 2B), while the level of LRRK2 in MO51EI+MO51IE group was statistically significant lower than that in control group (F(3, 8)  = 4.35, *P*<0.05). The aberrant transcript variants contained reading frame shifts and premature stop codons within the kinase or WD40 domain, implying the presence of putative truncated proteins.

### Knockdown of LRRK2 does not cause loss of DA neurons in zebrafish embryo

**Figure 3:**
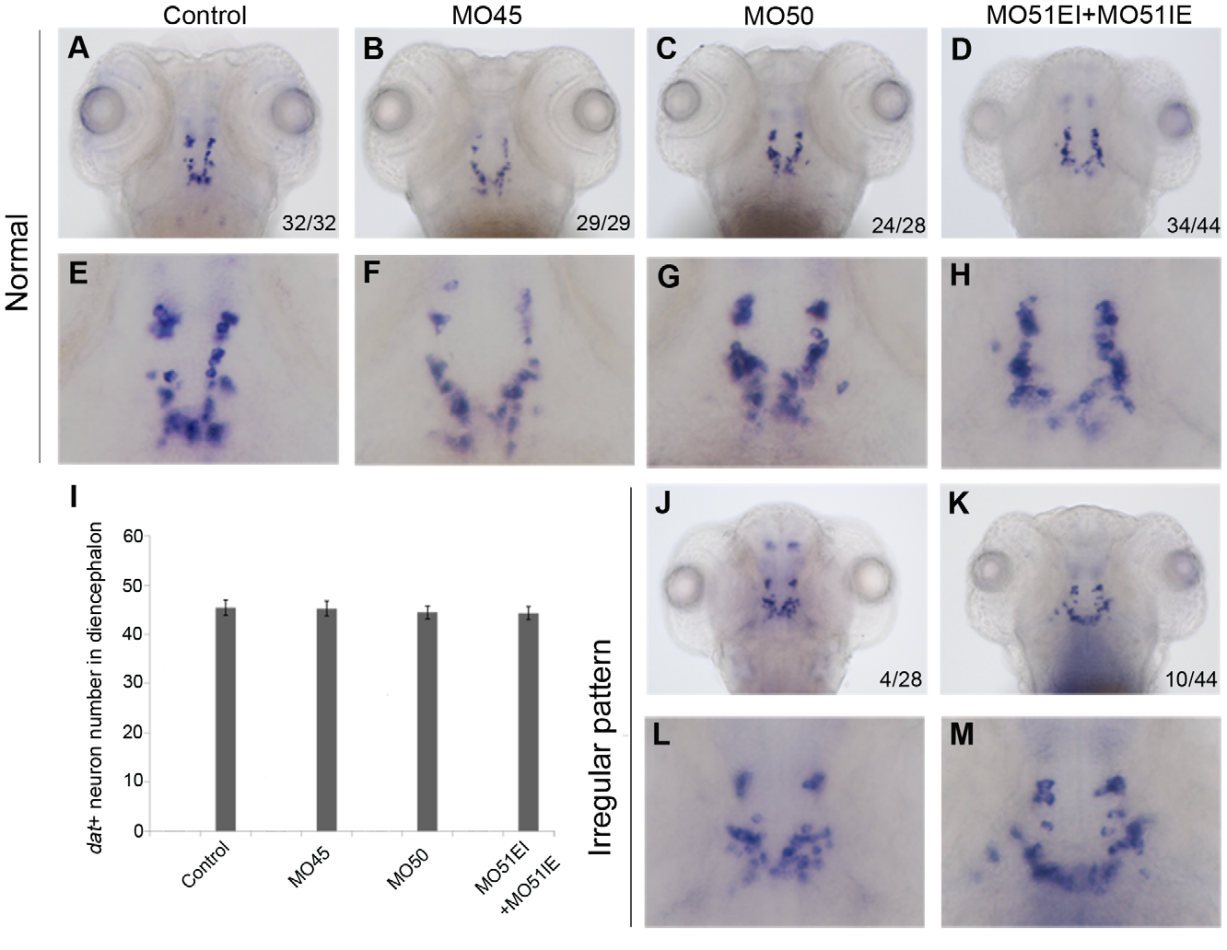
RNA whole mount *in situ* hybridization of 3 dpf embryos with *dat* probe didn't show significant loss of DA neurons in morphant. E to H, L and M, enlargement of the area of DA neurons in A to D, J, and K, respectively. The numbers on bottom right corner showed number of the embryos with a certain phenotype/number of total embryos in that group. The patterns of DA neurons in most embryos were normal, while some were disorganized. I, the quantitative result of *dat* positive neurons in the diencephalon. There is no significant DA neuron loss in morphant group. n = 20 in each group, *P*>>0.05 in all comparisons.

The MOs were injected into zebrafish embryos at a series of doses (Table 1). Most morphants developed 4-hour slower than un-injected control embryos without notable morphological defect at the dose of 8 ng per embryo or lower. We used 8 ng per embryo for experiments of this report since this concentration produced efficient block of LRRK2 mRNA splicing, as shown in Figure 2. At 3 dpf, *in situ* hybridization with *dat* (dopamine transporter) probe was performed to detected DA neurons[19]. For each of these MOs or MO combination, we did not observe loss of DA neurons in morphants (Fig. 3). Although MO50 and MO51IE+MO51EI caused some irregular patterns of DA neurons in diencephalons, specific loss of DA neurons was still not observed (Fig. 3J, K, L and M).

**Table 1:** Injection doses of morpholino oligos.

Morpholino oligos	Dose (ng per embryo)	Phenotype at 3 dpf
MO45	8, 10	8 ng group, 109 embryo alive, 12 embryo dead; 10 ng group, 148 embryo alive, 14 embryo dead. Live embryos developed about 4 hours slower than control.
MO50	6, 8, 10	6 ng group, 133 embryo alive, 16 embryo dead; 8 ng group, 108 embryo alive, 7 embryo dead; 10 ng group, 123 embryo alive, 14 embryo dead. Live embryos developed about 4 hours slower than control. In 10 ng group, 12 embryos had defects in head and heart.
MO51IE+MO51EI	8+8	129 embryo alive, 15 embryo dead. Live embryos developed about 4 hours slower than control.

**Figure 4:**
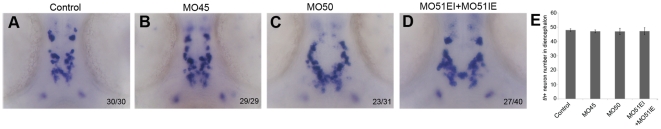
RNA whole mount *in situ* hybridization of 3 dpf embryos with *th* probe didn't show significant loss of DA neurons in LRRK2 morphants. A to D, the diencephalon region of zebrafish embryos, dorsal view, anterior to the top. The numbers on bottom right corner showed number of the embryos with a certain phenotype/number of total embryos in that group. There was not obvious alteration of DA neurons pattern in the diencephalon of the morphants (B, C, and D). E, quantative result of *th* positive neurons in the diencephalon. There was not significant DA neurons loss in all three morphant groups. n = 20 in each group, *P*>>0.05 in all comparisons.

In our studies of PD with zebrafish, we prefer using *dat* as probe to detect DA neurons since it is the most specific marker for DA neurons. However, Sheng *et al*. mainly used *th* (tyrosine hydroxylase) as probe to detect DA neurons in their report. To be consistent with their studies, we performed the same *in situ* hybridization using *th* probe and, again, no loss of DA neurons was observed in morphants (Fig. 4). There should be no difference between *dat* and *th* in detecting DA neurons because both of them are well established marker genes for PD studies [19], [20].

We also examined the locomotor phenotype of morphants and used their un-injected siblings as control. Locomotor experiments included tactile response test at 3 dpf and swimming ability assay at 6 dpf. Consistent with our *in situ* hybridization result with either *dat* or *th* probe, there were not obvious differences between control and morphant (Fig. S2).

Loss of function study of LRRK2 homologs in *C. elegans*, *Drosophila*, and mouse has been carried out. In *C. elegans*, *lrk-1* (LRRK2 homolog) regulates axonal-dendritic polarity of SV proteins, stress response and neurite outgrowth, but does not affect DA neurons[21], [22]. In *Drosophila*, two groups reported contrasting findings: Lee *et al*. reported that loss of LRRK (LRRK2 homolog) induced severe reduction of DA neurons[23], while Wang *et al*. reported that the LRRK mutant fruit fly developed normally without changing the number and pattern of DA neurons[24]. In LRRK2 knockout mouse, the dopaminergic system appeared intact in both young and aged mice. Furthermore, there was no significant difference in the susceptibility of LRRK2 knockout and wild type mice to MPTP[25]. Collectively, the findings in model animals suggest that LRRK2 plays a very limited role in the development or maintenance of DA neurons[7]. In conclusion, our study indicates that disruption of LRRK2 in zebrafish did not lead to loss of DA neurons. Considering the limitations of knockdown with antisense morpholino oligo and the importance of LRRK2 gene, to obtain a more solid conclusion of the function of LRRK2 in embryonic development and generation of DA neurons, we would like to employ engineered zinc-finger nucleases to inactivate LRRK2 in zebrafish in future study.

## Materials and Methods

### Zebrafish maintenance

Wild type AB was maintained in a circulating aquaculture system according to standard described in The Zebrafish Book[26]. Embryos were incubated at 28.5°C and staged according to the description by Kimmel et al[27]. At 24 hours post fertilization, 1-phenyl-2-thiourea (PTU, Sigma-Aldrich, St. Louis, MO,) was added to a final concentration of 0.003% to prevent the production of pigment. This zebrafish study was approved by Peking University Shenzhen Graduate School (09316).

### Design and injection of morpholino oligos

Five morpholino oligos were obtained from Gene Tools (Philomath, OR). They are: MO45, 5′CCCCTTCAGTATAAAAACACACTGT3′, targeting putative intron 44/exon 45 boundary; MO50, 5′AAATCTGCATGTTTTAGCACCTGGT3′, targeting putative exon 50/intron 50 boundary; MO51IE', 5′AGCTCCTGAAACACAGCATTAGGAA3′, targeting putative intron 50/ exon 51 boundary; MO51IE, 5′GCTCCTGAAACACAGCATTAGGAAC3′, targeting putative intron 50/ exon 51 boundary; MO51EI, 5′CACAA GCAGATTTATTAACCTGTGC3′, targeting putative exon 51/intron 51 boundary. These morpholino oligos were dissolved in RNase free ddH_2_O and microinjected by a PLI-90 microinjector (HARVARD APPARATUS, Holliston, MA) into one-cell fertilized egg. The volume of injection was calibrated using a glass capillary of 1 µl. The length of the glass capillary is 34 mm. By measuring the length of liquid injected (20 times) into the glass capillary, the volume per injection was thereby calculated, then the corresponding amount of MOs was also calculated. Injected embryos (morphant) were cultured in fish water at 28.5°C and allowed to grow up to 6 days.

### RT–PCR

Total RNA was isolated with RNAqueous^®^-4PCR Kit (Ambion, Austin, TX) from 3 dpf embryos and cDNAs were generated with PrimeScript™ RT reagent Kit (Takara, Dalian, China). Primers flanking target site were used to evaluate the efficacy of the each morpholino oligos. For MO45, the primers were F45-5′ GAGACGCTGCTGAAGAAA3′ (5386-5403) and R45-5′ CGAACTCACTGGGAAACT 3′ (6221–6238). For MO50, the primers were F50-5′ ATGTTTATTCGTTCGGTCTG3′ (6152–6171) and R50-5′ AGTGTCCCGTCTGCTGTG 3′ (6804–6821). For MO51IE', MO51IE and MO51EI, the primers were F51-5′TGCAAACGGAGGTAAAAACC3′ (6474–6493) and R51- 5′AGATGATCCTGGTCCCACAG3′ (6960–6979), which were the same as those used by Sheng et al[18]. The products were amplified by Taq PCR MasterMix (Tiangen, Beijing, China). The procedure of PCR was as following: 94°C for 5 min; 35 cycles of 94°C for 30 sec, 55°C for 30 sec and 72°C for 1 min; 72°C for 7 min; 16°C, forever. The PCR products were cloned into pGEM-Teasy vector (Promega, Madison, WI) for sequencing.

### Quantitative PCR

30 embryos of each group at 3 dpf were harvested. Total RNA was isolated with RNAqueous^®^-4PCR Kit (Ambion, Austin, TX) and cDNAs were generated with PrimeScript™ RT reagent Kit (Takara, Dalian, China). For LRRK2, the primers were as follows: 5′GACTCCGAGGCGATACAG3′ (forward, 778–795) and 5′CAAGGGCACTCAGACAGG3′ (reverse, 935–952); for beta-actin, the primers were as follows: 5′ GCCGTGACCTGACTGACTACCT3′ (forward) and 5′ CGCAAGATTCCATACCCAAGA3′ (reverse). Quantitative PCR was carried out on a 7300 real time PCR system (Applied Biosystems, Carlsbad, CA) with SYBR^®^ PrimeScript^®^ RT-PCR Kit (Takara, Dalian, China). Presented data were shown as mean±S.E.M of biological triplicates from at least two independent experiments and were presented as fold changes in relative expression as compared with control after being normalized to beta-actin. The relative fold changes were calculated by a comparative C_t_ method[28].

### Whole mount *in situ* hybridization and counting DA neurons

Digoxigenin-labeled anti-sense RNA probes were generated *in vitro* by using the zebrafish *dat* and *th* cDNA as templates with RNA polymerase (Promega, Madison, WI). Whole-mount RNA *in situ* hybridizations were performed essentially as described by Westerfield[26]. After *in situ* hybridization, transfer embryos into glycerol and equilibrate for 10 min. Then put it on a slide and flatten it softly with a cover slip to disperse the neurons (Fig. S1). The number of *dat* or *th* positive neurons in the diencephalon region was counted manually by two persons blinded to the knockdown of LRRK2.

### Imaging

Pictures of zebrafish embryos were taken with AxioImager A1 microscope and AxioCam digital camera (Zeiss, Oberkochen, Germany), and edited with Photoshop 7.0 (Adobe systems, San Jose, CA).

### Behavior assay

Tactile response assay of 3 dpf embryo was modified from Xi *et al.,* 2010[29]. Embryos in a 10 cm-diameter petri dish were put in the centre of a 3.5 cm field of a dissecting microscope (Zeiss, Oberkochen, Germany). Gently touched the tail of embryo with a forcep and recorded manually the times of touch to drive the embryo out of the field. Swimming ability assay of 6 dpf embryo was modified from Levin and Cerutti, 2009[30]. An embryo was put in a well (diameter = 3.5 cm) containing 3 ml fish water and being divided equally to 8 parts, accommodated for 10 min, then manually recorded the times of segment crossing to different parts by an embryo within 5min.

### Statistical analysis

All statistical analysis was performed with SPSS. The comparisons of differences between control and morphant groups were performed using one-way ANOVA. When the *P* value was less than 0.05, a Tukey HSD pairwise comparison post hoc test was conducted to determine the significance of difference between control and morphant groups. Error bars represented ± S.E.M. The number of used embryo was shown in the corresponding figure legend. All experiments were independently repeated at least three times. *P* values less than 0.05 were considered to be significant.

## Supporting Information

Figure S1 **Flatten embryo and count DA neurons.** Take embryos hybridized with *dat* probe as an example. After *in situ* hybridization, transfer embryos into glycerol and equilibrate for 10 min. Then put it on a slide and flatten it softly with a cover slip to disperse the neurons (A, dosal view; B, lateral view). C and D show the same embryo before and after being flattened.
(TIF)

Figure S2 **Behavioural assay showed morphant group embryos were normal in locomotor behavior.** A, result of tactile respone of 3 dpf embryo. n = 20 in each group, *P*>>0.05. B, result of swimming ability of 6 dpf embryo. n = 20 in each group, *P*>>0.05.
(TIF)
