# Supplementary figures and images for: Disruption of LRRK2 Does Not Cause Specific Loss of Dopaminergic Neurons in Zebrafish
Source: PLoS One. 2011 Jun 16;6(6):e20630. doi: 10.1371/journal.pone.0020630 (PMC3116841; doi:10.1371/journal.pone.0020630)

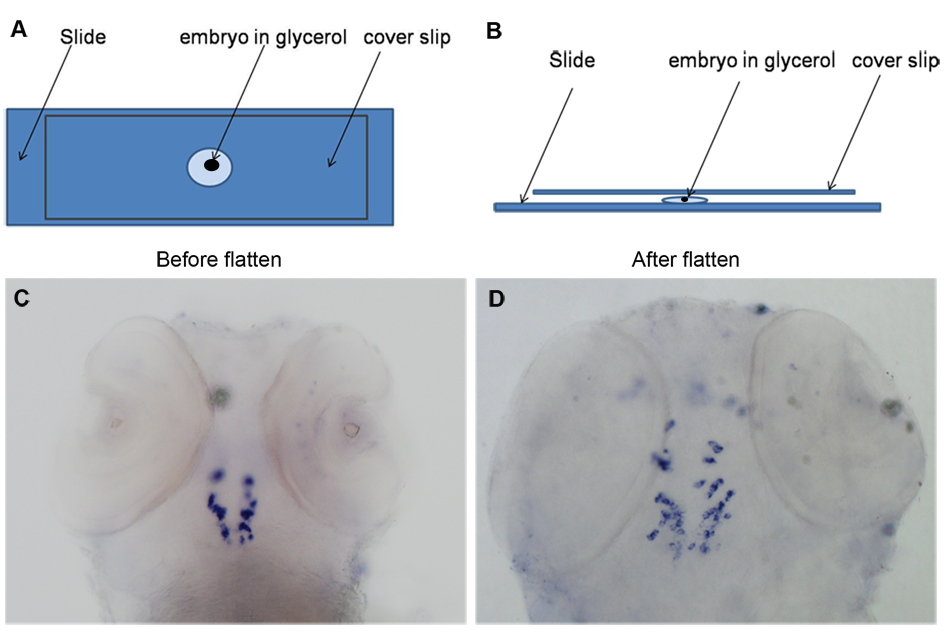

Supplement: Figure S1 — Flatten embryo and count DA neurons. Take embryos hybridized with dat probe as an example. After in situ hybridization, transfer embryos into glycerol and equilibrate for 10 min. Then put it on a slide and flatten it softly with a cover slip to disperse the neurons (A, dosal view; B, lateral view). C and D show the same embryo before and after being flattened. (TIF) [file pone.0020630.s001.tif]

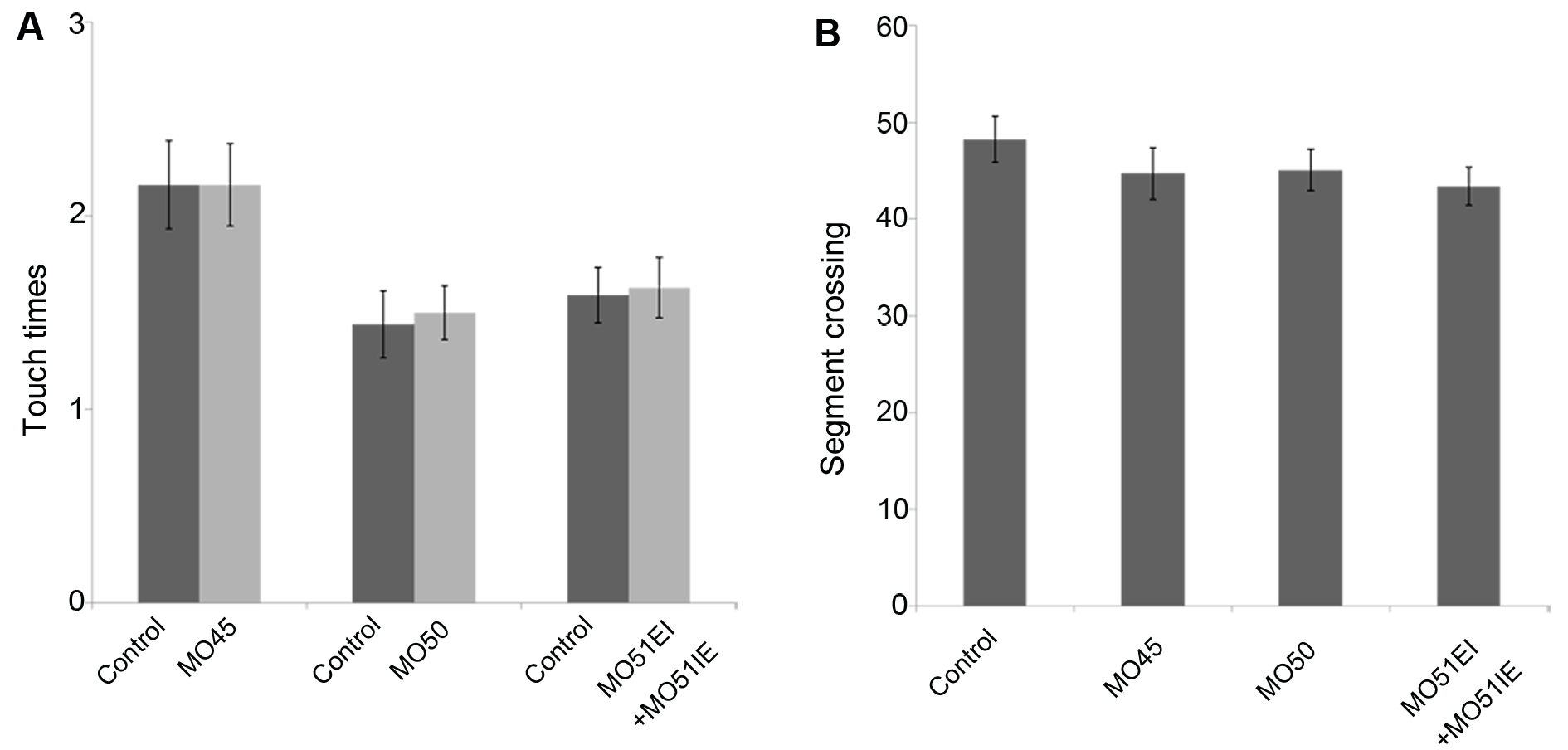

Supplement: Figure S2 — Behavioural assay showed morphant group embryos were normal in locomotor behavior. A, result of tactile respone of 3 dpf embryo. n = 20 in each group, P>>0.05. B, result of swimming ability of 6 dpf embryo. n = 20 in each group, P>>0.05. (TIF) [file pone.0020630.s002.tif]
